# Association between Pulse Pressure and Onset of Dementia in an Elderly Korean Population: A Cohort Study

**DOI:** 10.3390/ijerph17051657

**Published:** 2020-03-04

**Authors:** Yongku Jung, Dong-Woo Choi, Sohee Park, Sung-In Jang, Eun-Cheol Park

**Affiliations:** 1 Department of Health Policy and Management, Graduate School of Public Health, Yonsei University, Seoul 03722, Korea; andrew.jung2008@gmail.com; 2 Institute of Health Services Research, Yonsei University, Seoul 03722, Korea; CDW6027@yuhs.ac (D.-W.C.); jangsi@yuhs.ac (S.-I.J.); 3 Department of Public Health, Graduate School, Yonsei University, Seoul 03722, Korea; 4 Department of Biostatistics, Yonsei University Graduate School of Public Health, Seoul 03722, Korea; soheepark@yuhs.ac; 5 Department of Preventive Medicine, Yonsei University College of Medicine, Seoul 03722, Korea

**Keywords:** pulse pressure, dementia, elderly, women, men

## Abstract

*Objective:* There is paucity of studies on the association between pulse pressure and the development of dementia, although this association has already been established. This study aimed at investigating the association between pulse pressure and the onset of dementia. *Methods:* We used the South Korean National Health Insurance Service claims cohort data to select 149,663 patients without dementia aged ≥60 years. We calculated adjusted hazard ratios (HRs) and 95% confidence intervals (CIs) for dementia using Cox proportional hazard models according to a pulse pressure classification (<50, 50–59, 60–69, 70–79, 80–89, or 90+). *Results:* Compared to women with pulse pressure <50, those with pulse pressures of 50–59, 60–69, and 90+ had higher HRs for dementia (1.14, 1.22, and 1.03, respectively). These associations were particularly strong in those on Medicaid insurance and from rural regions. However, there were no statistically significant results among men. *Conclusions:* A higher pulse pressure was associated with an elevated risk of dementia in women aged >60 years, particularly those on Medicaid and from rural regions, possibly due to their inability to access hypertension and other medical treatment. The establishment of dementia indicators will help to guide future health policies for the prevention of dementia.

## 1. Introduction

Dementia is a major neuropsychiatric disorder, and is the fifth most common cause of death worldwide. Its prevalence has increased rapidly, leading to a high economic burden as a result of the growing social burden and costs related to the treatment to patients and their families [1]. Therefore, the early identification of factors that increase the risk of dementia is a critical task in terms of disease prevention, management, and policymaking [2]. For the early prevention of the onset of dementia, previous studies have identified several risk factors such as social, demographic, genetic, physical, and mental health factors [3,4,5]. 

Blood pressure is known to be an important indicator for predicting cardiovascular disease [6]. However, it is also recognized in the literature as a risk factor for dementia [7,8,9]. Elevated blood pressure likely to increase the risk for dementia by causing small-vessel disease and white-matter lesions [7]. A previous study found that middle-aged men with high blood pressure or hypertension had a higher risk for late age dementia than others who were treated [8]. One study, on the other hand, said that low blood pressure may be a complication of the dementia process, particularly Alzheimer’s disease [9].

Previous studies on hypertension have mainly focused on diastolic blood pressure in cognitive function and dementia and in the investigation of the relationship between a high blood pressure and the onset of dementia [10,11,12]. The Whitehall II cohort study found that 130 mmHg or above systolic blood pressure at age 50 is related to increased risk for dementia in individuals free of cardiovascular disease [10]. In contrast, another study showed that low diastolic pressure may be associated with a higher risk of the onset of dementia in the elderly, i.e., over age 75, due to cerebral hypoperfusion [11]. A systolic hypertension trial in Europe also found new evidence that blood pressure lowering therapy initiated with long-acting dihydropyridine protects against dementia in older patients with systolic hypertension [12]. High blood pressure is also strongly associated with a decline in cognitive function among the elderly [13,14]. One meta-analysis study found that hypertension has adverse effects on cognition, can alter cerebral vasculature integrity, and is associated with the pathogenesis of dementia [13]. In addition, people with high blood pressure were found to be likely to experience a cognitive decline in a relatively short time period; the risk is highest in untreated hypertensive patients [14]. 

While systolic and diastolic blood pressure have been reported as indicators of the onset of dementia in some studies, there are no consistent results regarding the relationship between pulse pressure and dementia [15,16,17,18]. Previous studies found that higher pulse pressure was an indicator that pulse pressure is associated with increased risk of dementia [15,16,17]. One cohort study showed that higher pulse pressure due to artery stiffness and severe atherosclerosis may be related to increased risk of Alzheimer disease and dementia in old adults [15]. A trial on hypertension in the very elderly found that wider pulse pressure may be an indicator of an increased risk of dementia; notably, they focused on the association between diastolic blood pressure and dementia [16]. Moreover, one longitudinal study revealed that increasing the level of pulse pressure and pulse wave velocity, which are markers of arterial stiffness, was related to the onset of dementia [17]. On the other hand, Freitag’s team showed that there is no association between pulse pressure and incidence of dementia [18]. Given these inconsistencies, this study investigated the association between pulse pressure and the onset of dementia.

## 2. Methods

### 2.1. Data

This study used data from the South Korean National Health Insurance Service-Health Screening Cohort (NHIS-HEALS) database. This dataset included information gathered on approximately 515,000 patients between 2002 and 2013 and represents a random sample of 10% of the patient cohort stratified according to age, sex, health insurance type, income level, individual total medical costs, and clinical test results. The data also included a list of diagnoses according to the International Classification of Diseases version 10 (ICD-10), medical costs claimed, prescribed drugs, and medical history [19].

### 2.2. Study Population

We conducted a cohort study using a random sample of national health insurance service claims data. We excluded patients diagnosed with dementia between 2002 and 2003. We also excluded patients with missing values for the covariates. From the 2004 to 2010 claims data, we selected patients without preonset of dementia who were aged ≥60. This resulted in a cohort of 149,663 patients. 

### 2.3. Variables

The onset of dementia was defined by the ICD-10 codes G30, G31.1, G31.9, G31.8, F00, F03, and F05.1. Blood pressure was measured using an automated sphygmomanometer during routine health screenings conducted every two years as part of a national health screening program. Pulse pressure was defined as follows: systolic blood pressure–diastolic blood pressure. This information was obtained from the NHIS-HEALS database. Pulse pressures were classified into six groups, namely, ≤50, 50–59, 60–69, 70–79, 80–89, and ≥90. 

Demographics and socioeconomic factors such as age, household income, and type of insurance were obtained from the NHIS-HEALS database. Patients were stratified into three groups based on their age as 60–69, 70–79, and ≥80 years. Patients were further stratified into quintiles based on their household income. In Korea, health care contributions are calculated according to household income. Patients who paid 0% of health insurance premiums were classified as Medicaid, those who paid up to 20% were classified as low income, those who paid 21–80% were classified as middle income, and those who paid over 80% of the premium were classified as high income. 

We used the Charlson comorbidity index (CCI) to measure comorbid conditions. The CCI assesses 19 diseases: myocardial infarction, congestive heart failure, peripheral vascular disease, cerebrovascular disease, dementia, chronic pulmonary disease, rheumatic disease, peptic ulcer disease, mild liver disease, diabetes without chronic complication, diabetes with chronic complication, hemiplegia or paraplegia, renal disease, any malignancy (including lymphoma and leukemia, except malignant neoplasm of the skin), moderate or severe liver disease, metastatic solid tumor, and AIDS/HIV. The CCI was calculated 1 year before patients were enrolled in the study [20]. The duration of treatment for hypertension (alpha- or beta-blockers, angiotensin converting enzyme inhibitors, angiotensin II receptor antagonists, diuretics, and calcium channel blockers), diabetes mellitus (sulfonylureas, metformin, thiazolidinedione, and alpha-glucosidase inhibitors), and dyslipidemia (any statins, fibrate, and omega 3 fatty acids) was determined using data on the duration of medications during the follow-up period. Hyperglycemia was defined as fasting blood glucose >110 mg/dL, measured in a clinical laboratory.

### 2.4. Statistical Analysis

We used descriptive statistics for the baseline characteristics of the study population. Differences between groups were determined using a chi-square test, t-test, and analysis of variance. Cumulative rates and incidence curves were constructed and compared using the Gray’s test for equality of cumulative incidence functions. Thereafter, we estimated the adjusted hazard ratios (HRs) and 95% confidence intervals (CIs) for dementia by applying a Cox proportional hazard regression analysis according to sex. In this model, we adjusted for systolic and diastolic blood pressure; age; region; household income; type of insurance body mass index (BMI); disability status; charlson comorbidity index (CCI); duration of treatment for hypertension, diabetes mellitus, dyslipidemia, and hyperglycemia; hospital classification; and year. Lastly, we conducted a subgroup analysis according to the type of insurance and region. All statistical analyses were conducted with SAS version 9.4 (SAS institute, Inc., Cary, NC, USA) and R version 3.6 with package “cmprsk” (R Core Team (2018). R: A language and environment for statistical computing. R Foundation for Statistical Computing, Vienna, Austria. URL: https://www.R-project.org/). Statistical significance was set at *p* < 0.05.

## 3. Results

Table 1 shows baseline demographic characteristics of the study population. Among 149,663 patients, 73,272 were men and 76,391 were women. The rates of pulse pressure in the six groups (<50, 50–59, 60–69, 70–79, 80–89, and ≥90) were 1.6%, 1.8%, 1.9%, 1.9%, 2.2%, and 2.3%, respectively, in men and 2.5%, 2.7%, 3.1%, 3.1%, 3.5%, and 4.5%, respectively, in women with the onset of dementia. The total number of men and women with dementia was 1.8% and 2.7%, respectively. According to age groups (60–69, 70–79, and ≥80 years), the percentages of participants were 70.0% (*n* = 51,303), 27.7% (*n* = 20,051), and 2.6% (*n* = 1918) in men, 67.3% (*n* = 51,449), 29.8% (*n* = 22,764), 2.9% (*n* = 2178) in women respectively.

**Figure 1:**
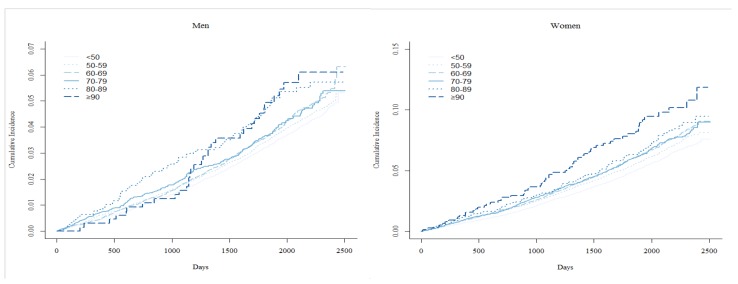
The cumulative incidence function of dementia using the Gray’s test for pulse pressure.

Figure 1 shows the cumulative incidence function of dementia using the Gray’s test for pulse pressure. The cumulative incidence curve for the group of patients with pulse pressures of ≥90 was lower than that of other groups in both men (*p* = 0.01) and women (*p* < 0.0001).

Table 2 shows the adjusted HRs for dementia using Cox proportional hazard models for pulse pressure and covariates. Among women, the 50–59 (HR: 1.14, 95% CI: 1.04–1.25), 60–69 (HR: 1.22, 95% CI: 1.07–1.39), and ≥90 (HR: 1.03, 95% CI: 1.03–1.80) pulse pressure quartiles showed higher HRs for the incidence of dementia than the ≤50 quartile. However, there were no statistically significant differences for pulse pressure in men.

Table 3 shows the adjusted HRs for the subgroup analysis. For women in the Medicaid group, the 50–59 (HR: 5.71, 95% CI: 2.02–16.12), 60–69 (HR: 9.16, 95% CI: 2.31–36.36), 70–79 (HR: 17.53, 95% CI: 3.23–95.12), 80–89 (HR: 19.91, 95% CI: 3.59–110.56), and ≥90 (HR: 86.80, 95% CI: 11.76–1.640.69) pulse pressure quartiles were associated with higher HRs for the incidence of dementia than the ≤50 quartile. However, there were no statistically significant results for men. For women who lived in the capital area, the 50–59 and 60–69 pulse pressure quartile groups had higher HRs than the ≤50 group. For women who lived in rural areas, only the ≥90 pulse pressure group had a higher HR than the ≤50 group.

## 4. Discussion

This study examined the association between pulse pressure and the onset of dementia among men and women. After controlling for several covariates, elderly women with a high pulse pressure were shown to have a significantly higher rate of onset of dementia than those with a low pulse pressure. Moreover, these associations were strong among women in the Medicaid group who lived in rural areas. 

The results from this study showed that high pulse pressure levels were associated with the risk of developing dementia. These results are similar to those from previous studies that reported the association between an elevated pulse pressure and decreased cognitive function in community members aged 55–64 years using data from the Framingham Offspring Cohort Study conducted to identify risk factors for cardiovascular disease during the 1998–2009 period [21]. In addition, previous studies have demonstrated an association between an increased pulse pressure and pulse rate and decreased cognitive function [16,22]. The association between high pulse pressure levels and an increased risk of developing dementia may be explained by the traditional belief that dementia is a cerebral neurodegenerative disease that occurs as a result of the accumulation of β-amyloid, as well as the method of reporting and interpreting it as a result of cerebral white matter changes due to vascular diseases [23]. On the other hand, more recent studies offer evidence for the role of the disconnection hypothesis of Alzheimer’s disease due to white matter lesions of the frontal-prefrontal cortical-subcortical circuits accounting for cognition and mood-affect [24,25,26,27,28]. These findings have been supported using transcranial magnetic stimulation and transcranial Doppler sonography.

Another possibility is that pulse pressure has a high positive correlation with plasma B-type natriuretic peptide (BNP), and high BNP levels have a high correlation with microvascular damage. Therefore, damage and disease in the microvessels of the cerebrum cause the denaturation of blood vessels. These negative changes in the blood vessels destroy the function of the blood–brain barrier, thereby increasing the accumulation of β-amyloid due to increased permeability and protein runoff in the cerebral cortex [29,30,31,32,33].

In contrast, atherosclerosis, a vascular disease, of the blood vessels leads to a rise in the blood pressure. Vascular rigidity and atherosclerosis increase the risk of dementia, indicating that vascular diseases impose a similar risk as cardiovascular disease [34,35,36]. It has also been reported that blood vessel-rigidity can cause microvascular disorders and diseases in the brain [37]. Rigidity reduces the absorption of waves in the arteries, leading to an increased pressure in blood flow to the capillaries of the brain. These disorders of the cerebral blood vessels can increase the white matter in the brain, which is common in patients with dementia [37]. Therefore, pulse pressure and the range of white matter lesions in the brain and their severity are highly relevant. White matter abnormalities are also highly related to cognitive impairment [38,39,40,41,42]. It can be inferred that high pulse pressure levels are associated with increased brain white matter lesions and the occurrence of dementia through microvessel disorders in the cerebrum [38,39,40,41,42]. Therefore, those diseases are likely to be synergistic effects to developing dementia [43]. Moreover, one study found the possibility that pulse pressure may be also related to increased cerebrospinal fluid and phosphorylated-tau in normal elderly [44]. It is possible that pulsatile hemodynamics may be associated with amyloidosis and tau-related neurodegeneration [44].

In this study, the association between a high pulse pressure and the high risk of developing dementia was evident only in women. This observation was similar to that of previous studies conducted in elderly [16,45]. One study found that lower pulse pressure was 1.4 times more common among women than men [45]. This study proposed that the association between pulse pressure and dementia is due to artery stiffness and severe atherosclerosis, and poor cerebral perfusion [45]. Another study also found that women were 1.5 times more likely than men to have a probability of Alzheimer’s disease and severity of cognitive impairment [16]. Although we could not find the underlying mechanisms for the difference between sexes, we guess that it is possible that women often receive less education, and are subject to the stronger effect of Apolipoprotein E, and bilateral oophorectomy compared to men [46]. In contrast, depressive symptoms may affect the increasing risk of dementia in elderly women. Many studies have found that women are more likely to be with depressive symptoms than men [47,48,49,50]. This may be due to differences in genetic influence and the social role of women, e.g., as caregivers, living alone, and social support [47,48,49,50]. Depressive symptoms could be related to dementia. A previous study mentioned that vascular depression may also be related to white matter lesions due to vascular damage to frontal subcortical circuits [38]. Therefore, these factors may have led to the high incidence of dementia in elderly women. However, we could not take into account the proportion of depressive symptoms among both men and women at baseline, which may have affected the results. Despite these studies, medical or biological explanations for the elevated risk of dementia in women remain controversial due to a paucity of evidence; therefore, further research is needed.

Our study had several limitations. First, because the follow-up period from the medical examination to the onset of dementia took place over a 10-year-period, it is possible that we under- or over- estimated the link between the measurement of blood pressure and fasting blood glucose depending on the time of the examination or the current health condition of the patient. In addition, we could not include patients who did not undergo blood pressure testing or a health check-up every 2 years. However, blood pressure was measured at a clinical laboratory; therefore, the measurements were reliable. Second, although we used the ICD-10 codes for the diagnosis of dementia, this might have been overestimated due to up-coding errors or misclassification biases associated with the claims data. Therefore, the results should be interpreted with caution. Finally, there is an added potential for the overestimation or undervaluation of statistical results due to unadjusted confounding factors.

## 5. Conclusions

Although our findings need further confirmation, we believe that there is an association between pulse pressure and the incidence of dementia. A higher pulse blood pressure is associated with an elevated risk of dementia in individuals aged >60 years, but only in women. Furthermore, the results from the subgroup analysis revealed that elderly women with Medicaid living in rural areas should be targeted so that interventions aimed at preventing dementia can be implemented among this population. 

## Figures and Tables

**Table 1:** Baseline characteristics of the study population from 2004 to 2010.

Variables	Total	The Number and Incidence of Dementia				
		Men		Women		*p–Value*
		*N*	%	*N*	%	
Pulse pressure						0.0493
<50	57,608	944	1.6	1428	2.5	
50–59	49,610	872	1.8	1344	2.7	
60–69	27,046	515	1.9	831	3.1	
70–79	10,595	201	1.9	329	3.1	
80–89	3367	74	2.2	117	3.5	
≥90	1437	33	2.3	64	4.5	
Systolic blood pressure						0.5369
<120	50,755	852	1.7	1403	2.8	
120–129	11,917	177	1.5	270	2.3	
130–139	39,039	687	1.8	1007	2.6	
>140	47,952	923	1.9	1433	3.0	
Diastolic blood pressure						0.2388
<60	2046	31	1.5	62	3.0	
61–80	58,020	1005	1.7	1604	2.8	
81–90	55,127	955	1.7	1486	2.7	
>90	34,470	648	1.9	961	2.8	
Age						< 0.0001
60–69	102,752	1269	1.2	2001	1.9	
70–79	42,815	1212	2.8	1873	4.4	
80+	4096	158	3.9	239	5.8	
Region						0.0135
Capital area	46,295	684	1.5	894	1.9	
Metropolitan area	34,280	553	1.6	832	2.4	
Rural	69,088	1402	2.0	2387	3.5	
Household income						0.0099
1st quintile	29,273	528	1.8	941	3.2	
2nd quintile	22,894	444	1.9	628	2.7	
3rd quintile	22,747	374	1.6	520	2.3	
4th quintile	30,458	493	1.6	733	2.4	
5th quintile	44,291	800	1.8	1291	2.9	
Types of insurance						0.0003
NHI, self–employed insured	148,839	2622	1.8	4055	2.7	
Medical aid	824	17	2.1	58	7.0	
BMI						<0.0001
Underweight (<18.5)	70,488	125	0.2	166	0.2	
Normal (18.5–22.9)	44,447	1141	2.6	1401	3.2	
Overweight (23–24.9)	21,981	681	3.1	1024	4.7	
Obesity (≥25)	12,747	692	5.4	1522	11.9	
Disability						0.0361
Yes	2419	112	4.6	64	2.6	
No	147,244	2527	1.7	4049	2.7	
CCI						0.0210
0	70,488	1162	1.6	1529	2.2	
1	44,447	786	1.8	1379	3.1	
2	21,981	390	1.8	782	3.6	
3+	12,747	301	2.4	423	3.3	
Duration of treatment for hypertension						0.0937
0	45,413	630	1.4	709	1.6	
1–365 days	71,503	1193	1.7	2208	3.1	
366–730 days	21,096	506	2.4	824	3.9	
>730 days	11,651	310	2.7	372	3.2	
Duration of treatment for diabetes mellitus						1.0000
0	116,896	1982	1.7	3038	2.6	
1–365 days	20,851	345	1.7	635	3.0	
366–730 days	7303	188	2.6	287	3.9	
>730 days	4613	124	2.7	153	3.3	
Duration of treatment for dyslipidemia						0.2083
0	89,894	1644	1.8	2089	2.3	
1–365 days	36,571	480	1.3	1150	3.1	
366–730 days	14,350	294	2.0	579	4.0	
>730 days	8848	221	2.5	295	3.3	
Hyperglycemia						0.1142
No	116,463	1998	1.7	3203	2.8	
Yes	33,200	641	1.9	910	2.7	
Hospital classification						0.0298
General hospital	39,371	680	1.7	971	2.5	
Hospital	34,314	653	1.9	1,068	3.1	
Clinic	75,978	1306	1.7	2074	2.7	
Year						0.0540
2004	46,217	1033	2.2	1429	3.1	
2005	50,882	990	1.9	1558	3.1	
2006	21,264	348	1.6	586	2.8	
2007	13,830	176	1.3	330	2.4	
2008	7331	59	0.8	130	1.8	
2009	6808	28	0.4	67	1.0	
2010	3331	5	0.2	13	0.4	
Total	149,663	2639	1.8	4113	2.7	

Note: BMI, Body mass index; CCI, Charlson comorbidity index; NHI, National health insurance.

**Table 2:** Adjusted hazard ratio for dementia using Cox proportional hazard models.

Variables	Hazard Ratio of Dementia			
	Men		Women	
	HRs	95% CIs	HRs	95% CIs
Pulse pressure				
<50	1.00		1.00	
50–59	1.00	(0.89–1.13)	1.14	(1.04–1.25)
60–69	0.99	(0.84–1.16)	1.22	(1.07–1.39)
70–79	0.91	(0.73–1.12)	1.14	(0.96–1.35)
80–89	1.03	(0.78–1.36)	1.15	(0.92–1.44)
≥90	1.03	(0.70–1.50)	1.37	(1.03–1.80)
Systolic blood pressure				
<120	1.00		1.00	
120–129	0.91	(0.77–1.08)	0.85	(0.74–0.98)
130–139	1.00	(0.87–1.15)	0.83	(0.74–0.93)
>140	1.02	(0.83–1.24)	0.83	(0.70–0.97)
Diastolic blood pressure				
<60	1.00		1.00	
61–80	1.13	(0.79–1.62)	1.08	(0.84–1.40)
81–90	1.02	(0.71–1.48)	1.13	(0.86–1.47)
>90	1.00	(0.68–1.48)	1.14	(0.86–1.51)
Age				
60 – 69	1.00		1.00	
70–79	2.33	(2.14–2.53)	2.11	(1.98–2.25)
80+	3.50	(2.95–4.16)	3.49	(3.03–4.01)
Region				
Capital area	0.73	(0.67–0.81)	0.71	(0.65–0.77)
Metropolitan area	0.82	(0.75–0.91)	0.85	(0.78–0.92)
Rural	1.00		1.00	
Household income				
1st quintile	1.08	(0.96–1.21)	1.04	(0.95–1.13)
2nd quintile	1.09	(0.97–1.23)	0.97	(0.88–1.07)
3rd quintile	1.00	(0.88–1.13)	0.87	(0.78–0.96)
4th quintile	0.97	(0.86–1.08)	0.90	(0.82–0.99)
5th quintile	1.00		1.00	
Types of insurance				
NHI, self–employed insured	0.91	(0.56–1.47)	0.59	(0.45–0.77)
Medical aid	1.00		1.00	
BMI				
Underweight (<18.5)	0.85	(0.70–1.02)	1.11	(0.94–1.30)
Normal (18.5–22.9)	1.00		1.00	
Overweight (23–24.9)	0.88	(0.80–0.97)	0.93	(0.85–1.00)
Obesity (≥25)	0.83	(0.76–0.92)	0.87	(0.80–0.93)
Disability				
Yes	1.41	(1.16–1.70)	1.23	(0.96–1.58)
No	1.00		1.00	
CCI				
0	1.00		1.00	
1	1.12	(1.02–1.22)	1.16	(1.08–1.25)
2	1.10	(0.98–1.23)	1.28	(1.17–1.39)
3+	1.26	(1.11–1.43)	1.27	(1.13–1.41)
Duration of treatment for hypertension				
0	1.00		1.00	
1–365 days	1.40	(1.26–1.55)	1.29	(1.18–1.41)
366–730 days	1.85	(1.59–2.16)	1.70	(1.48–1.95)
>730 days	1.94	(1.57–2.41)	1.50	(1.21–1.85)
Duration of treatment for diabetes mellitus				
0	1.00		1.00	
1–365 days	1.02	(0.89–1.16)	1.24	(1.12–1.36)
366–730 days	1.21	(1.01–1.46)	1.22	(1.05–1.41)
>730 days	1.09	(0.86–1.37)	1.21	(0.99–1.49)
Duration of treatment for dyslipidemia				
0	1.00		1.00	
1–365 days	1.14	(1.02–1.27)	1.04	(0.96–1.13)
366–730 days	1.08	(0.91–1.28)	1.10	(0.96–1.26)
>730 days	1.22	(0.97–1.53)	1.09	(0.87–1.36)
Hyperglycemia				
No	1.00		1.00	
Yes	0.97	(0.88–1.07)	0.94	(0.87–1.02)
Hospital classification				
General hospital	0.98	(0.89–1.08)	1.01	(0.93–1.09)
Hospital	1.05	(0.96–1.16)	1.14	(1.05–1.22)
Clinic	1.00		1.00	
Year				
2004	1.00		1.00	
2005	1.15	(1.04–1.26)	1.06	(0.98–1.15)
2006	1.48	(1.30–1.69)	1.22	(1.10–1.35)
2007	1.60	(1.35–1.90)	1.43	(1.26–1.62)
2008	1.61	(1.22–2.11)	1.79	(1.48–2.16)
2009	1.62	(1.10–2.39)	1.84	(1.43–2.38)
2010	1.84	(0.76–4.48)	2.06	(1.18–3.59)

Note: BMI, Body mass index; CCI, Charlson comorbidity index; NHI, National health insurance.

**Table 3:** Adjusted hazard ratio for incidence of dementia according to subgroups using Cox proportional hazard models *.

Subgroups	Pulse Pressure	Hazard Ratio of Dementia			
		Men		Women	
		HRs	95% CIs	HRs	95% CIs
Types of insurance					
NHI, self–employed insured	<50	1.00		1.00	
	50–59	1.00	(0.89–1.13)	1.12	(1.02–1.24)
	60–69	0.98	(0.84–1.16)	1.20	(1.05–1.37)
	70–79	0.90	(0.73–1.12)	1.11	(0.93–1.31)
	80–89	1.00	(0.76–1.33)	1.11	(0.88–1.39)
	≥90	1.03	(0.71–1.51)	1.28	(0.97–1.71)
Medical aid	<50	1.00		1.00	
	50–59	0.81	(0.12–5.55)	5.71	(2.02–16.12)
	60–69	1.07	(0.06–19.99)	9.16	(2.31–36.36)
	70–79	4.12	(0.11–151.70)	17.53	(3.23–95.12)
	80–89	7.88	(0.31–199.00)	19.91	(3.59–110.56)
	≥90			86.80	(11.76–640.69)

Note: NHI, National health insurance; Analysis was adjusted for the following covariates: systolic blood pressure, diastolic blood pressure, age, region, household income, BMI, CCI, duration of treatment for hypertension, diabetes mellitus, dyslipidemia, hyperglycemia, hospital classification, and year.
